# Participant Experiences of a COVID-19 Virtual Clinical Study Using the Current Health Remote Monitoring Platform: Case Study and Qualitative Analysis

**DOI:** 10.2196/37567

**Published:** 2022-07-05

**Authors:** Juliana Pugmire, Jessie Lever Taylor, Matt Wilkes, Adam Wolfberg, Nicole Zahradka

**Affiliations:** 1 Current Health Ltd Edinburgh United Kingdom; 2 Current Health Inc Boston, MA United States

**Keywords:** virtual trial designs, virtual enrollment, digitalized health, theoretical domains framework, thematic analysis, remote patient monitoring

## Abstract

**Background:**

During the COVID-19 pandemic, individuals with a positive viral test were enrolled in a study, within 48 hours, to remotely monitor their vital signs to characterize disease progression and recovery. A virtual trial design was adopted to reduce risks to participants and the research community in a study titled Risk Stratification and Early Alerting Regarding COVID-19 Hospitalization (RiskSEARCH). The Food and Drug Administration–cleared Current Health platform with a wearable device is a continuous remote patient monitoring technology that supports hospital-at-home care and is used as a data collection tool. Enrolled participants wore the Current Health wearable device continuously for up to 30 days and took a daily symptom survey via a tablet that was provided. A qualitative substudy was conducted in parallel to better understand virtual trial implementation, including barriers and facilitators for participants.

**Objective:**

This study aimed to understand the barriers and facilitators of the user experience of interacting with a virtual care platform and research team, while participating in a fully virtual study using qualitative and quantitative data.

**Methods:**

Semistructured interviews were conducted to understand participants’ experience of participating in a virtual study during a global pandemic. The schedule included their experience of enrollment and their interactions with equipment and study staff. A total of 3 RiskSEARCH participants were interviewed over telephone, and transcriptions were inductively coded and analyzed using thematic analysis. Themes were mapped onto the Theoretical Domains Framework (TDF) to identify and describe the factors that influenced study adherence. Quantitative metrics, including adherence to wearable and scheduled tasks collected as part of the RiskSEARCH main study, were paired with the interviews to present an overall picture of participation.

**Results:**

All participants exceeded our definition of a fully adherent participant and reported that participation was feasible and had a low burden. The symptoms progressively resolved during the trial. Inductive thematic analysis identified 13 main themes from the interview data, which were deductively mapped onto 11 of the 14 TDF domains, highlighting barriers and facilitators for each.

**Conclusions:**

Participants in the RiskSEARCH substudy showed high levels of adherence and engagement throughout participation. Although participants experienced some challenges in setting up and maintaining the Current Health kit (eg, charging devices), they reported feeling that the requirements of participation were both reasonable and realistic. We demonstrated that the TDF can be used for inductive thematic analysis. We anticipate expanding this work in future virtual studies and trials to identify barriers and enabling factors for implementation.

## Introduction

### Background

With the onset of the COVID-19 pandemic in 2020, we have seen rapid shifts in the way people work, engage with education and health care, and conduct their activities of daily life [1]. Many traditional clinical trials have slowed to a halt because of health care shortages and fear of increasing viral transmission [2]. Studies involving human participants have adapted to better use digitalized, decentralized, or virtual trial designs by the end of 2020 (though perhaps not as drastically as expected) [3]. Similar to remote working, virtual trial designs were a possibility that existed before the pandemic but have become a necessity for many researchers wanting to reduce the risk of transmission in human participants and the research community alike, while still conducting research [4,5].

Virtual clinical trials (VCTs) are site-less and rely on technologies such as apps, web-based platforms, wearable devices, and remote monitoring [6]. Digitized clinical trials also use technology to recruit and retain participants and for data collection and analysis [7]. Digitized clinical trials or VCTs leverage digital health technologies to improve participant access and engagement [7-9]. These trial designs have the potential to lower the cost of these studies and expand participation by making trials more accessible to participants [9,10].

With the shift to virtual, digitalized clinical trial designs, it may be helpful for study participants to understand specific implementation issues, including barriers and facilitators. Recruitment and retention in clinical trials are persistent challenges, whether traditional or virtual [7,11,12]. In VCTs, the study participant will likely have to interact with technology they may not have previous experience with, such as a remote continuous monitor, new apps for e-consenting and tracking, or daily surveys delivered by tablets [13,14]. There will almost certainly be a learning curve, with any instruction or assistance available also delivered remotely. Besides technical barriers, there may also be concerns about participant privacy when it comes to sharing sensitive health information [9].

### Current Health and Risk Stratification and Early Alerting Regarding COVID-19 Hospitalization

Current Health (Current Health Ltd, Edinburgh, United Kingdom) is a medical technology company that creates a platform that enables continuous remote patient monitoring to support hospital at home programs and care [15]. The Food and Drug Administration–cleared Current Health kit includes a wearable device, which is a small, round disk that is attached to a band and worn on the upper arm. It monitors respiration rate, heart rate, oxygen saturation, skin temperature, and activity [15]. It can be integrated with peripheral devices, including those measuring blood pressure, axillary temperature, spirometry, weight, and continuous glucose. It also incorporates a tablet that can deliver surveys, reminders to take measurements (eg, blood pressure, weight), or a video connection to a health care provider or investigator. It requires approximately 5 minutes for a participant to set up the Current Health kit, including measuring and selecting the correct arm band size, and begin transmitting vital sign data via the secure wireless home hub. The home hub allows the Current Health platform to operate without an in-home Wi-Fi connection, thereby making the technology more inclusive.

The Current Health platform was used in the study, Risk Stratification and Early Alerting Regarding COVID-19 Hospitalization (RiskSEARCH; NCT04709068) [16], funded by the US Department of Health and Human Services branch, the Biomedical Advanced Research and Development Authority. Its purpose was to remotely monitor individuals who tested positive for COVID-19 infection, within the previous 48 hours, to learn more about disease progression and recovery. The enrolled participants wore the Current Health wearable device continuously for up to 30 days. Health data were collected to develop predictive models for the risks of hospitalization and death.

As part of the main study, the research team designed a qualitative substudy run in parallel to gain an in-depth understanding of the participant’s experience of taking part in a virtual study. Participants first had to show that they were eligible for the study by answering a web-based eligibility questionnaire, chose a time to connect with a study coordinator to be consented and enrolled, and finally had to set up and use the Current Health kit, which was shipped to their home address, all without meeting the study personnel in person. Once enrolled, participants were asked to answer a daily symptom survey delivered via a tablet and wear the Current Health wearable device 24 hours a day, except when charging the device or showering, bathing, or swimming. For the substudy, participants also agreed to conduct an interview of up to 40 minutes about the experience of participating in the RiskSEARCH study and using the Current Health kit.

The RiskSEARCH study did not progress beyond the pilot phase because of the changing landscape of the COVID-19 pandemic, including vaccine development and receding waves of infection, which negatively affected recruitment [17]. However, the substudy collected in-depth data on 3 participants, presented here as a case series, and qualitative analysis applying the Theoretical Domains Framework (TDF) to better understand the participant experience.

### Theoretical Domains Framework

Virtual studies such as RiskSEARCH have many components that demand behavioral adaptation to adhere to the study intervention (eg, engaging in specific ways with the Current Health platform). The TDF synthesizes 128 theoretical constructs from 33 theories into a combined theoretical framework comprising 14 domains [18]. The TDF has been used to evaluate implementation problems, understand the mechanisms of change, and design interventions. The TDF helps researchers identify and describe the factors that influence a set of behaviors (eg, study adherence). More specifically, it can help investigate implementation issues, including barriers and facilitators, to participating in studies such as the RiskSEARCH study and adopting the behavior changes necessary for adherence.

This is an exploratory piece of research based on a virtually delivered study run during the global COVID-19 pandemic from March 2021 to May 2021. The study team conducted this research to explore the participant experience for improving (1) recruitment and retention in future studies, (2) user experience with the Current Health platform, and (3) the ease with which the platform can be harnessed in other clinical studies, and in particular, virtual studies. We hope that these findings will aid other investigators to successfully conduct virtual studies and VCTs.

## Methods

### The RiskSEARCH Main Study

The RiskSEARCH study was a virtual, time-sensitive trial for individuals, aged >21 years, who tested positive for COVID-19 infection. The primary purpose of this study was to develop a machine learning–based algorithm to predict the likelihood of requiring a hospital stay of at least 24 hours using data collected from a remote patient monitoring wearable device and symptom surveys. This study used the Current Health platform for hospital-grade remote patient monitoring of vital signs and daily symptom surveys. Participants were recruited through advertisements on social media (Facebook, LinkedIn, etc) and word of mouth from March 2021 to May 2021. If an individual met the inclusion and exclusion criteria (Multimedia Appendix 1) and were interested in participating, they had 48 hours to enroll in the study. They were then consented and shipped a Current Health kit. The details of the main study will be published in a separate paper.

Each day, the participants were sent a 21-question survey to complete on the Current Health tablet. The survey asked if participants experienced 8 specific symptoms (chills, fever, nausea, diarrhea, sore throat, dry cough, muscle ache, and loss of smell or taste) and whether they were better, worse, or the same as the previous day. In addition, there was a free-text response in which participants could add any other symptoms they were experiencing. Questions were also included about whether participants were likely to contact a health care provider or attend a hospital based on how they felt that day. This symptom survey was developed and piloted internally before it was shared with the RiskSEARCH study participants. Its purpose was to capture the symptoms and symptom severity associated with COVID-19 infections, to help drive the prediction model of the main study. In parallel, participants were asked to wear the Current Health wearable device for up to 30 days, taking it off only to charge (up to 30 minutes every 24 hours), shower, bathe, or swim.

### Qualitative Substudy

We used semistructured interviews and reported the results following the consolidated criteria for reporting qualitative research checklist [19]. Specifically, we wanted to understand what it was like to use the Current Health kit and participate in a fully remote virtual study during a global pandemic. We collected in-depth data on the acceptability of the RiskSEARCH study and Current Health kit. Focused qualitative and quantitative research provided insights into the user experience of interacting with the Current Health kit, the Current Health research team, enrollment process, and participation in a fully virtual study.

### Research Objectives

The research objectives of this study are presented in Textbox 1.

**Research objectives.**

**Research objectives**

- To explore recruitment and retention for the Risk Stratification and Early Alerting Regarding COVID-19 Hospitalization (COVID-19) study
- To explore the feasibility, acceptability, and usability of the intervention, that is, the Current Health wearable device and tablet
- To explore barriers and facilitators of study compliance

### Topic Guide and Interviewing

On the basis of the literature, our research objectives, and previous experience in developing interviews to understand engagement with digital technology, the study team designed an interview schedule (Multimedia Appendix 2) to explore barriers and facilitators around different aspects of the study and intervention (web-based enrollment, answering the daily survey, charging the wearable device, etc). One-to-one interviews were conducted by JP via telephone at a prearranged, mutually convenient time. JP was a senior clinical research scientist at Current Health at the time of this study, has >10 years of experience conducting interviews for qualitative and mixed methods research, holds a Doctor of Public Health and Master of Public Health in epidemiology, and is a woman.

### Recruitment and Procedure

Although we planned to use a purposive sampling strategy, we changed to convenience sampling when the main study recruitment remained low. A total of 7 participants were offered the opportunity to participate in a one-to-one interview with a research team member (JP). Participants were approached by the study coordinator (JLT) through text messaging or telephone conversations after building rapport through the study enrollment process. A total of 4 participants agreed to participate in the study, and 3 interviews were conducted. A participant could not be contacted to set up the interview. No relationship was established between the interviewer and the interviewee before the commencement of the study. The participants knew that JP was a research scientist at Current Health and was interested in understanding their experience of participating in RiskSEARCH and using the Current Health kit.

Participants who agreed to participate in the interview were sent a PDF version of the informed consent form (ICF). Participants were sent the ICF via DocuSign (DocuSign, Inc) 24 hours before the interview. Participants could sign ahead of the call with the researcher or wait until the call to complete the ICF and ask any questions before signing. The researcher (JP) ensured that the participant questions were answered and that the participants understood the risks of study participation. Participants could opt out of recording the interviews, but none chose this option.

### Intervention

Once enrolled in the main study, participants were required to wear the Current Health device at all times, except when charging the device (20-30 minutes every 24 hours) or when showering, bathing, or swimming. They were also required to keep the tablet charged and answer the daily symptom surveys delivered by the wearable device. Textbox 2 shows the components of the intervention.

**Components of the intervention.**

**Components**

- Be home to receive the Current Health kit delivered by FedEX
- Open Current Health box
- Set up home hub which includes plugging hub into the wall
- Select correct armband size using included sizing guide (out of 3 sizes)
- Charge wearable device on included dock until fully charged, indicated by green lights, and charge daily thereafter
- Insert wearable device into armband and wear next to skin under clothing
- Remove wearable device for showering, bathing, or swimming
- Charge tablet daily
- Answer daily symptom surveys delivered on the tablet
- At the completion of the main study (up to 30 days), repackage the Current Health kit back into the box and use the return label provided to arrange return
- For substudy, arrange a mutually convenient time to be interviewed
- Participate in an over-the-phone interview lasting up to 40 minutes about using the Current Health kit and participating in the Risk Stratification and Early Alerting Regarding COVID-19 Hospitalization study

### Data Collection

Participant interviews were conducted over telephone and audio recorded using a laptop application (Windows Voice Recorder, Microsoft Corporation) and a handheld digital recorder as a backup. Interviews were anonymized and transcribed using Trint software (Trint Ltd) and checked, corrected, and edited for accuracy by the researcher who conducted the interviews (JP). Familiarization with the data began at this early stage. Participants were also asked to take a modified Telehealth Usability Questionnaire (TUQ) sent to them via an email link. The TUQ is a validated survey tool that quantifies the usability of telehealth implementations and services [20]. No repeat interviews were carried out, no field notes were made, transcripts were not returned to participants for correction, and participants did not provide feedback on the findings.

### Metrics

As part of the main study, interview participants also contributed quantitative data, such as daily symptom surveys submitted via tablets. The data collected relevant to the substudy included the following variables as shown in Textbox 3.

The participants’ symptoms and vital sign alarms were presented alongside the qualitative results, as their clinical course may have influenced their experiences.

**Data collected.**

**Variables**

1. Wearable adherence: the time the wearable device was worn compared with the study duration.
2. Daily survey adherence: the number of daily surveys completed compared with the number of daily surveys assigned.
3. Fully adherent, determined using 3 criteria: wearables worn for at least 20 hours a day and at least 6 days a week up to 30 days, daily survey responses at least 6 days a week up to 30 days, and a returned Current Health kit at the end of study participation.
4. Vital signs alarms: alarm thresholds were set for vital sign data going out of range, which could only be seen by the study team.

### Analysis

A researcher (JP) conducted the interviews, transcribed the audio recordings using Trint transcription software, and coded the data using NVivo Qualitative Data Analysis Software (version 12; QSR International) [21]. We used reflexive thematic analysis [22]. Data were analyzed inductively following the steps of Braun and Clarke [22,23], specifically (1) familiarization of data, (2) generating initial codes, (3) searching for themes, (4) reviewing themes, (5) defining and naming themes, and (6) producing the report. Initial codes were inductively generated from the interview transcripts, iteratively condensed, and expanded into themes. The themes were then deductively mapped onto the domains of the TDF.

### Ethics Approval

Ethics approval was obtained from the Institutional Review Board, Advarra (Columbia, Maryland, ethics approval number Pro00047371). The collected data were stored in compliance with the European Union General Data Protection Regulation, Current Health Research Data Management Policy, US Health Insurance Portability and Accountability Act, and Current Health Research Data Management Policy. Data were anonymized, and all personal identifiers were removed.

## Results

### Participant Characteristics and Quantitative Results

#### Overview

Participant details are provided in Table 1 and discussed in further sections.

**Table 1:** Participant characteristics.

Participant ID	Gender	Age (years), range	Wearable adherence (%)	Daily survey adherence (%)	Telehealth Usability Questionnaire score
RS001	Female	30 to 35	83	76	7
RS006	Female	40 to 45	63	90	—^a^
RS008	Female	35 to 40	92	100	—

^a^Participants did not complete the Telehealth Usability Questionnaire.

#### Case 1

**Figure 1:**
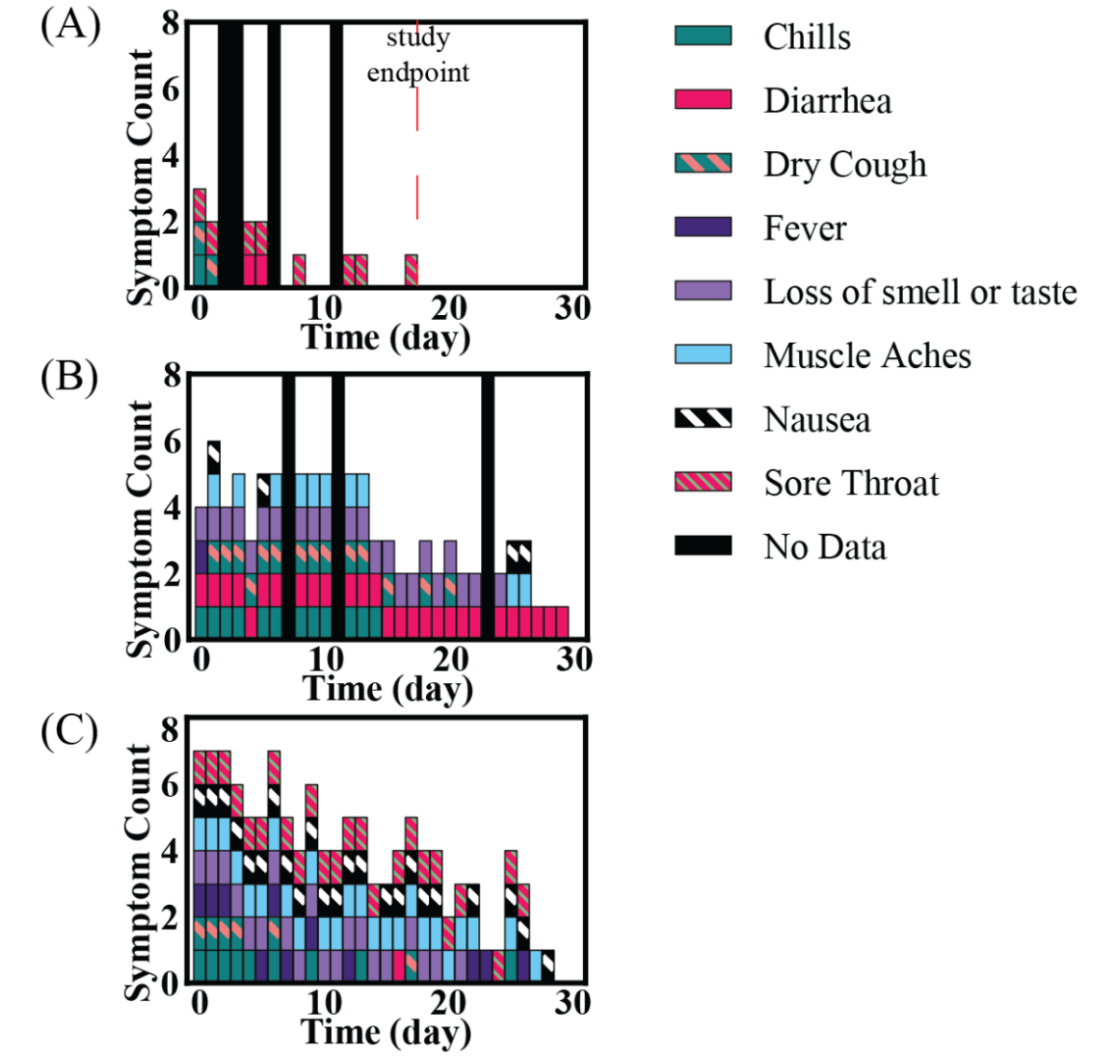
Symptom survey data for participants, daily symptoms reported by participants: (A) RS001, (B) RS006, and (C) RS008. Reported symptoms varied by participants. White gaps between days indicate the participant reported feeling no symptoms. Black bars indicate the days that the participant did not complete the daily symptom survey. Red hatched line indicates the study duration ended before 30 days.

RS001 initially reported experiencing chills, dry cough, and a sore throat. She did not report experiencing any other symptoms for the duration of her study. By day 6, RS001’s chills and dry cough resolved and did not reoccur. However, she reported a sore throat periodically throughout her 17 days in the study. (Figure 1A) Over the course of the study, the only vital sign that triggered an alarm on the Current Health dashboard was a high respiration rate, which occurred on days 0, 3, and 9.

#### Case 2

RS006 experienced all 8 symptoms, specifically asked about in the daily symptom survey over the course of the study. Her fever resolved on day 1 and did not reoccur. Conversely, her diarrhea did not resolve until day 30. Her symptoms decreased over the 30 days of study participation (Figure 1B). This trend in self-reported symptoms aligned with her vital signs data. RS006 triggered 34 alarms, but no alarms were triggered from day 23 onward. Nearly half of the alarms triggered were for a high pulse rate with a low amount of motion detected (ie, the participant’s pulse rate was high while not exerting themselves physically). Other alarms triggered were for low oxygen saturation and a high respiration rate with a low amount of motion detected.

#### Case 3

RS008 experienced all 8 symptoms included in the daily symptom survey, although diarrhea was reported only once on day 16. Nausea was the most persistent symptom, continuing until day 28. No symptoms were reported on days 29 or 30. As with the other participants, RS008’s symptoms improved over the course of the study (Figure 1C), which was also reflected in her vital signs data. RS008 triggered 10 alarms during the 30-day study period. No alarms were triggered after day 19.

Although all participants were asked to follow a link provided via email to complete the TUQ survey, only one participant completed the survey. This participant scored *strongly agree* (7 on the 7-point Likert scale) to all 21 questions of the TUQ, indicating high levels of usefulness, ease of use, effectiveness, reliability, and satisfaction with the Current Health kit.

### Qualitative Results

#### Overview

In all, 3 interviews were conducted toward the end of study participation when participants were feeling better. Interviews ranged from 18 to 35 minutes. Inductive thematic analysis helped identify 13 main themes and subthemes associated with the participant experience of using the Current Health kit and being part of the RiskSEARCH study and included (1) Participant Situations, (2) Getting Started, (3) Study Support, (4) Study Communication, (5) Protecting and Contributing, (6) Determination, (7) Study Pros & Cons, (8) Optimism, (9) Uncertainty, (10) Payment, (11) Accessing Data, (12) Memory & Reminders, and (13) Making Habits.

These themes were deductively mapped to the TDF domains. These domains are described below and presented in Table 2. There were 3 domains of the TDF in which we did not match any data to: *Intentions*, *Goals*, and *Emotion*.

The main domains of the TDF, which we were able to map our themes onto, were *Environmental Context and Resources*; *Knowledge* combined with *Skill*; *Social/Professional Role and Identity*; *Beliefs about Capabilities*; *Optimism*; *Beliefs about Consequences*; *Reinforcement*; *Memory*, *Attention*, *and Decision Processes*; *Social Influences*; and *Behavioral Regulation*.

**Table 2:** Theoretical Domains Framework (TDF) constructs, Risk Stratification and Early Alerting Regarding COVID-19 Hospitalization (RiskSEARCH) themes, and description.

TDF constructs	RiskSEARCH themes	Description
Environmental context and resources	Participant Situations: being sick with COVID-19 infection; caretaking responsibilities	Study participants were recruited and went through the study after testing positive for COVID-19 infection Study required steps that may have been more challenging for participants who had many caretaking responsibilities
Environmental context and resources	Getting Started: enrollment; kit components; for example, wearables; unknowns; suggestions	Participants had to self-navigate through a web-based enrollment system and website Current Health kit required setup by the participant themselves (though they did have access to technology and study team support) Communication between tablet or wearable and participant Wearable needed charging 30 minutes every 24 hours. Participant did not know the battery level of the wearable, but green lights on charger indicated that it was fully charged Suggestions for improving any aspect of the Current Health kit
Knowledge combined with skill	Study Support^a^: personnel; materials	Technology support was available 24/7 to help with any aspect of setting up or using the wearable The study team was available on demand to answer any questions relating to the study or Current Health kit The Quick Start Guide was available in a hard-copy booklet in the Current Health kit or digitally accessible via the tablet “Study Support’ double coded with Social Influences”
Knowledge combined with skill	Study Communication^a^: passive; active	Website as a source for information on the study and COVID-19 pandemic “‘Study Communication’ double coded with Social Influences”
Social or professional role and identity	Protecting and Contributing	Help or protect others; feeling a sense of community responsibility; wanting to help in a difficult time; being someone who helps Motivation to help others A sense of contributing toward the management of the COVID-19 pandemic
Beliefs about capabilities	Determination	The level of commitment while engaging with the Current Health kit—survey or tablet or wearable
Optimism	Study Pros and Cons	Positive and negative aspects of the study
Optimism	Optimism	Seeing the positive in the bad situation of being tested positive for COVID-19 infection
Beliefs about consequences	Uncertainty	Feedback regarding user’s “performance” or whether kit was working properly
Reinforcement	Payment	Study participants expressing their views on the US $100 offered for their time and effort
Reinforcement	Accessing Data	Having access to own data The wearable device does not transmit data to the participant
Memory, attention, and decision processes	Memory and Reminders	Remembering to charge and wear the wearable Reminders to take the survey every day Reminders to charge the tablet and take the survey
Social influences	Study Support^a^: personnel	Possibility to contact technology support or study team “Double coded with Knowledge combined with Skill”
Social influences	Study Communication^a^: active	Via email, text, or telephone call “‘Study Communication’ double coded with Knowledge combined with Skill”
Behavioral regulation	Making Habits	Habit formation around charging the wearable and tablet and taking the survey Ability to support routine or habit formation

^a^Mapped to 2 different domains from the TDF.

#### Environmental Context and Resources

Many contextual factors impacted the participants’ ability to successfully participate in the RiskSEARCH virtual study, including the Current Health kit functionality and design, being sick with COVID-19 infection, caretaking responsibilities, and comfort of the wearable device.

First, they had to navigate through a web-based enrollment process that included clicking on an advertisement that took the participant to a brief eligibility screening questionnaire and onto an appointment booker to connect with a member of the study team for consent. Although 2 participants said that the enrollment process was smooth and easy, another participant reported some minor problems that required contact with the study team:

I think it was pretty easy.

RS008

So when I went to enroll, it didn’t give me a time slot, like it said that there was no one available. I guess you have to like, talk to someone at first and I remember it led me - it led me all the way to the end. But then I said, like, there was no - no one available for my initial call... So I emailed and then they were available. But it was like, still on the same day. I feel like it was a glitch or something.

RS001

Second, participants were required to set up the Current Health kit that was delivered to their home, select the correct-sized armband, charge the device and tablet, insert the device into the armband, and begin wearing it. In all, 2 participants described the setup as an easy process with a participant providing negative feedback on the tablet stand, which they described as nonessential and fussy:

Because like, one of the first things in the instructions is...take out the stand, put the...um...put the tablet on the stand. Like I just said, you could take all of that out because at first I thought I had to do it for it to get started and I didn’t, and it wasn’t like standing up and it just seemed like a waste.

RS001

It was super easy in the box set up that, you know, getting the tablet and everything and then getting the little charging dock. And I mean, it was easy and then I got it connected to my wifi and started wearing it that day.

RS006

The third participant experienced problems during setup. The Current Health kit shipping was delayed, and when she began setting it up, there was a problem with connectivity, which she reported took her a few hours to work out:

And then once I got the stuff here, yeah I started setting it up and then either the mobile or the wifi wouldn’t work...it just didn’t want to connect the wifi or wearable device.

RS008

Through the process of enrollment and setting up the wearables, participants were sick with COVID-19 infection, which meant that the usual barriers to joining a study may have been more difficult than usual. As a study team, we attempted to make the process as easy as possible for the participants:

It [enrollment] was super easy to me. I mean, even while, you know, sick as a dog with COVID I was still able to navigate and do it. So if I was able to, then you know, anybody could as long as you read and understand what you read, you can do it.

RS006

Furthermore, study adherence may have been more difficult for participants who had caretaking responsibilities:

It’s hard when you’re a caretaker and you’ve got, you know, your mom with breast cancer. So you have to keep her schedule up plus her meds. Plus your schedule and your meds and then hubby and his meds and his schedule. It can get overwhelming, I guess, but it was just because I probably was out of my routine.

RS006

Adherence could also be influenced by the comfort of the wearable device, which we asked participants to wear as close to 24/7 as possible, only removing the wearable device to shower, bathe, swim, or charge. They could switch arms but needed to wear it next to their skin, under their clothes. In all, 2 participants said that it was comfortable and did not give them any problems, even during sleep. A third participant provided suggestions for improvement:

Maybe if the band was...it could get...more air towards it, so you won’t get so much sweat under it...It’s you know, really gross with activity sometimes...so maybe just more airing.

RS008

#### Knowledge Combined with Skill

The domains of Knowledge and Skills were combined. Participants had to acquire an understanding of how to use the wearable device and tablet for adherence. Participants were provided with a printed and digital version of the QuickStart Guide (QSG) in the Current Health box and on the tablet. All participants reported using the hard copy QSG, which was positioned to be very visible upon opening the box, and none of the participants were aware that the QSG was also available on the tablet:

I remember getting everything [Current Health kit] and then I just - as soon as you open the box, I mean, like literally step by step, as long as you follow that booklet inside. That’s what I did. I read it first. And then I started looking at stuff and I went back and I was like, OK, step 1 is this, step 2 is this. I mean, it hooked up in like literally ten minutes.

RS006

When a participant encountered problems with setup or connection, they had access to the study team and Current Health’s 24/7 technical support to get things working:

I mean, I couldn’t connect, of course. Then finally I was like, OK...then I realized I could reach out to the email person or the person that was like head of tech things like and say, OK, it’s not working.

RS008

An important aspect of technical support is the speed at which they respond, so that the participants do not become frustrated and no data are lost:

...they contacted me pretty fast, so...I didn’t think I was going to have a response like that.

RS008

Participants had to develop skills to interact with the tablet to take daily symptom surveys and to ensure that the tablet and wearable device were charged and working. They also had to experiment with the device fit to ensure comfort:

It’s [survey] very, very easy to understand straight to the point, like you ask exactly what you need to know. And I love how it gets to all the symptoms, you can hit yes or no. And then it even asks you okay is it better than the day before this time or worse. I have loved that because some of my symptoms are a lot better and some of them are staying worse or getting worse and it varies daily. And so, yeah, I love that. That’s really cool.

RS006

Now, if you’ve got the band too tight on your arm, your arm will hurt. That’s a learning process whenever you’re starting. I did that...Then I got it loosened up and it’s like perfect now.

RS006

#### Social or Professional Role and Identity

The participants talked about their reasons for joining the study and contributing their time and effort. They were motivated to help in what few ways they felt they could, especially when it was difficult to help beyond isolation at home. Having a sense of contribution to efforts around the COVID-19 pandemic is important:

Look, I’m trying to be a good...I’m trying to be a good human. We’re trying to quarantine and stay away from people.

RS006

...well, I’ll apply and see and help out the community and help out the hospital or where all this data was going to go to help you guys. See if it would do anything good for covid.

RS008

#### Beliefs About Capabilities

The participant who had problems with the setup of the Current Health kit showed particular determination in working through the issues and troubleshooting until she could get it working. Although she had access to technical support, she was determined to troubleshoot initial connection problems independently:

And then once I got the stuff here [Current Health kit], I started setting it up and then either the mobile or the wi-fi wouldn’t work... I tried doing stuff on my own... it just didn’t want to connect to the wi-fi or wearable device…I was like OK, I’m not going to play with this anymore. And then stayed up, like all night cause I was like OK, I am not letting this thing beat me. I was just determined to like…I’m going to figure this out somehow and then yeah... I don’t even know what time it was, I don’t know, it was like twelve or one-o’clock in the morning when I finally got it to work.

RS008

#### Optimism

A participant showed tremendous optimism in the face of the COVID-19 pandemic and her own personal trouble in being sick with the disease. This participant focused on what it was teaching her and helping her focus on gratitude for aspects of life that were going well:

It’s [COVID] definitely teaching us and it’s making me learn and making me more aware and I’m thankful for it if you wanna know the truth.

RS006

Yeah, that’s...that’s a positive way to look at things, huh?

Interviewer

Yes, ma’am. I mean, well, you got to be positive. I was already down to the bottom. You know, I was already at the bottom of the COVID barrel.

RS006

Although we did not hear that our participants expressed pessimism in relation to the COVID-19 pandemic or their involvement in the study, we asked them if there were any negative aspects to participating in the study. The participants mentioned that charging and remembering to charge the device and tablet were inconvenient tasks. A participant said that you could become tired of wearing the device, but she did not mind wearing it. Wearing the device could also pique the curiosity of strangers:

The only negative thing is people ask, what is that on your arm?

RS006

Oh, interesting.

Interviewer

Yes, that would be I would say the only thing, just getting questions about what is it? What are you wearing? And so I just tell them, I’m in a medical study since I had COVID and I’m just giving all my data and vitals and having stuff recorded. That’s what I tell them.

RS006

#### Beliefs About Consequences

The Current Health platform at the time of this study did not relay any information to the participants. It took some time for participants to adjust to wearing and charging the device and trusting that the data were being transmitted appropriately. Participants did not always know if data would be lost if they left their homes. The biggest issue that came up for participants around charging the device was not knowing how much battery it had left, making it difficult to plan charging the device. The tablet was less of an issue because they could leave it on the charger and only use it once per day to take and submit their daily symptom survey. Several suggestions have been made to make the battery life more apparent to the user, which are now being integrated into the product:

I don’t know if it’s possible, but like if it told you if you needed to charge your device. Like I know it tells you, please recharge it but if there’s like a way it told you that it had a low battery the actual like...I have no idea if it’s possible but like, if it somehow would like, oh, your little arm band has low battery, charge it.

RS001

So I guess, you know, I wasn’t sure if like, it would work if I wasn’t in my house.

RS001

After a power outage, a participant expressed concern over the lost data and whether the device was still transmitting the data:

I was having an issue about the connection and I emailed [the Study Coordinator] and I was like, are you getting this [vital signs data] and she said, Yes, you’re fine, you’re good, it’s okay. Cause we had like a power outage so our Internet went out and everything. And I was like, Are you still getting this? Yes, it holds data for eight to ten hours. I said, Okay, just making sure because I thought I’d done messed it up [laughs].

RS006

We found this domain to be linked with a concern that participants had about their own study adherence (ie, transmitting vital signs data) and desire to participate in the study to the best of their ability.

#### Reinforcement

A participant felt that the study would be more engaging if she could see some of her own data, and plans are underway to allow participants and patients to receive feedback on their data from wearing the device.

Participants were asked what they thought about the US $100 they were offered for the time and effort they took to participate in the study. Participants received this payment after the study ended and the equipment was returned to Current Health. All participants thought it was a nice gesture but said it was not the thing that motivated them to participate:

I think it’s fine, like it didn’t...it didn’t sway me to participate or not participate...It’s just a nice added bonus.

RS001

I think it’s more than fair...I mean, all you’re doing is just wearing this device. It’s not like you’re having to drive anywhere. You’re not having to go out of your way. All you have to do is hook up some equipment, wear the device and save the box. And then when you’re done, pack it all up and send it in. Woo hoo, I mean. It is not that hard. So I mean, I think it’s very fair.

RS006

I think it’s like a nice thing...I didn’t do it for the money, I mean, I’m still waiting on that.

RS008

#### Memory, Attention, and Decision Processes

The participants in the RiskSEARCH study had high levels of adherence to wearing the device and taking the daily survey (see the aforementioned results). For the few times they did not answer the symptom survey or wear the device, we asked them to think about the reasons. A major contributor to not wearing the device was forgetting to put it back on after removing it for charging, bathing, or showering. For the tablet, it was forgetting to take the survey:

Yes, I think I forgot to put it on...And then other than that, I don’t think I...I did stop it early because I went on vacation and I didn’t bring it with me [participant asked to stop participating before going on vacation as symptoms had resolved].

RS001

I think I pretty much wore it all the time. Sometimes...like whatever going to the shower or whatever, then maybe I...might've like left it on the charger a little bit longer.

RS008

#### Behavioral Regulation

Participants spoke about the importance of routine and habits for remaining adherent by wearing a charged device and having a charged tablet to take daily symptom surveys. On days when a participant was out of routine or when normal habits could not be completed, there was an increased risk of forgetting to do these things:

I was literally running all day long from home like 9:00 that morning until about 5:30 yesterday afternoon is when I finally stopped and got home. And when I came in and made something to eat, I didn’t even...I got, I was out of my routine that day. And I didn’t even think about it. I didn’t even think about it until three o’clock this morning.

RS006

#### Social Influences

Participants had access to the Current Health technical support 24/7 and the study team close to 24/7. It was critical that we not lose participants or otherwise good adherence because of problems or questions that could not be responded to quickly. The participants could access technical support over telephone and the study team over telephone, email, or text. Participants spoke highly about the study’s clinical research associate who was primarily responsible for enrolling participants and helping them get set up.

The lady that I was coordinating with was...she was super sweet, she was super informative. Anytime I had a question all I had to do with text or message, and she literally got back to me that same day.

RS006

We also looked for feedback about study aspects, such as communication, frequency of messaging, and the Current Health kit itself. We asked in-depth questions about their experience using the study website, study-related emails, texts, and telephone calls. Participants found the methods of communication acceptable and unobtrusive and said that they liked having several avenues available to them for communication with the study team.

## Discussion

### Principal Findings

To gain a more thorough understanding of participant experience in a fully remote virtual trial during a global pandemic, semistructured interviews were conducted with 3 of the 7 participants in the RiskSEARCH study. All participants met our criteria for being fully adherent to the study and reported through interviews or the TUQ that participation requirements in both the main study (up to 30 days of wearing a wearable device on the upper arm, responding to daily surveys, and communicating with the study team when necessary) and the substudy were feasible and low burden.

Having quick and easy access to support from the study team and Current Health technical support was a critical enabling factor for staying engaged [24]. Future virtual studies should ensure that this resource, a dedicated and responsive study team or technical support, is accessible to participants (Textbox 4). This may necessitate staffing across time zones or during out-of-office hours. Participants reported some barriers around setting up the Current Health kit, keeping devices charged, and remembering to take surveys but described these as minor challenges and showed high adherence while wearing the device and submitting responses to daily symptom surveys. Combining subjective (qualitative and self-report) and objective (quantitative like the number of surveys submitted and hours of vital signs data transmitted) data, the researchers assumed that high adherence was at least partly tied to ease of participation. A participant reported high levels of adherence (ie, reported not wearing the device on only 2 to 3 occasions that were a few hours long), while objectively showing 63% wearable device adherence. In reviewing the data, we believe that some data may have been lost when the participant was away from the home hub for >8 hours. Several factors may contribute to the differences in objective and subjective measures when collecting remote data such as perception, unknown technical issues, or improper use of digital technology. We found that the overall motivation for enrolling was a wish to contribute positively to the COVID-19 effort. In this small sample, we found adherence to be the easy part, whereas the key challenge for the research team was recruitment to the main study amidst the rapidly shifting landscape of the pandemic.

**Recommendations.**

**Recommendations**

- Provide participants quick and easy access to support from the study team or technical support for any digital health technologies used in the study.
- Collect in-depth information around factors that impact on study enrollment and adherence; for example, processes, communications with study team, advertisements, and trouble setting up or using technology.
- Identify discrepancies in subjective and objective measures of study adherence and try to understand why those might exist; for example, participant perception, unknown technical issues, or improper use of digital technology.
- Identify agreement in subjective and objective measures of study adherence to understand what is working well.
- Consider using the Theoretical Domains Framework (or similar framework) for assessing potential implementation problems in virtual trials.

The interview schedule (Multimedia Appendix 2) was developed with the purpose of understanding the participant’s experience of interacting with the Current Health kit and the ability and motivation to adhere to the study intervention. This interview schedule can be used in other qualitative studies looking to identify components of the study, such as digital technology and study materials, that facilitate or prevent adherence. We explored the barriers and facilitators to the web-based study enrollment process, communication with the study team, study advertisements and recruitment messaging, troubleshooting, burden of participating in the study, ease of use of the Current Health kit, and benefits and disadvantages of participating in the RiskSEARCH study. The data were then inductively coded into themes related to the TDF domains. The TDF is frequently used to develop interview schedules, with interviews coded into 14 domains of the TDF. The authors could not find research conducted as it was for this study, with an interview schedule developed independently of the TDF with themes from analyzed interview data and then mapped onto the TDF.

The TDF worked well for our qualitative data and the process of mapping inductive themes onto domains from the TDF was relatively straightforward. We chose the TDF because it was developed to make behavior change theories more accessible to implementation researchers [18]. It was revised and validated in 2005 [25] to help researchers identify and describe the barriers and facilitators that could influence behavior and thereby impact implementation. Evidence suggests that theoretically based health interventions are more successful than interventions with no such base [26]. We needed a method for theoretically assessing any potential implementation problems encountered while running the RiskSEARCH study; the TDF provided this method. We are unaware of the use of TDF in other virtual studies or VCTs.

The interview schedule, created independently of the TDF, produced themes that mapped most heavily onto the domains *Environmental Context and Resources* and *Knowledge* combined with *Skills*. We believe this is an indication of the critical aspects of the product itself, the built environment of the participants, and the knowledge and skill acquisition that are essential for setting up and using the Current Health kit.

There were 3 domains that we did not map any themes to: *Intentions*, *Emotions*, and *Goals*. Though we did not map any themes to the TDF domain of *Intentions* it was clear throughout each interview that participants made a conscious effort to be fully adherent by wearing the device as long and as often as possible, answering the daily symptom survey, and returning the Current Health kit once the study was over. As for *Emotions*, we found that participants were content to wear the device and be adherent. For the one participant who had trouble setting up her Current Health kit, she did not describe feeling frustrated or annoyed but simply determined to get her Current Health kit working without assistance, although knowing that technical support was available. This was not a study designed to get participants to create and follow goals, which is why we likely did not have any themes that could be mapped to the domain *Goals*. In future interview schedules, we could consider targeting these domains to obtain the most complete picture of implementation using the TDF.

### Limitations

The biggest limiting factor in this study was the sample size. Although “data saturation” is a term with some conceptual and methodological issues and is not a necessity in every type of qualitative research [27,28], this study would have benefited from more interviews and in particular from interviewees who were different ages, male, and not adherent or those who experienced technical challenges, troubleshooting, and had opportunities to develop strategies around device charging and remembering to complete the daily symptom survey. There is also a possibility that more interviews would have led to more themes that could have been mapped onto the 3 domains of the TDF, for which we did not have data. However, reaching saturation does not necessarily invalidate our findings [29]. Despite the low number of interviews, we believe these exploratory findings add value to identifying barriers and facilitators to adherence in virtual studies and specifically, studies that require using wearables and submitting daily digitally delivered surveys.

### Future Work

We hope to expand these preliminary findings to future virtual studies and VCTs that will surely outlive the current COVID-19 pandemic [30]. As a study team, we are highly motivated to reduce the burden placed on study participants to make study adherence as easy and enjoyable as possible and to encourage a more diverse study population by removing logistical barriers to participation [31]. The findings from this exploratory research will contribute to the best-practices literature for VCTs and help improve the Current Health kit and study delivery for future research participants. We believe that there is also more to learn about motivating factors for participants willing to enroll and participate in infectious disease research. We are also developing a means of providing the participant’s own data to them to help with engagement and memory around charging and wearing the device and believe that this will affect adherence metrics and overall levels of study engagement.

As the COVID-19 pandemic stretches on and the need for VCTs continues to grow, there is also a need for continuing research that helps us understand the participants’ experience of engaging in these studies, including the barriers and enabling factors that influence adherence. The RiskSEARCH study did not progress beyond the pilot study because of limited recruitment, highlighting an ongoing need to improve recruitment for clinical studies, whether virtual or in person. Despite the problems with recruiting, we believe we have learned some critical lessons about the conduct of virtual study or trial presented in this paper and have produced tools to continue collecting this type of data.

### Conclusions

Participants in the RiskSEARCH substudy showed high levels of adherence and engagement throughout their participation. Although participants experienced some challenges in setting up and maintaining the Current Health kit (eg, charging devices), they reported feeling that the requirements of participation were both reasonable and realistic. We have shown that the TDF can be used for inductive thematic analysis. We anticipate expanding this work in future virtual studies and trials to identify barriers and enabling factors for implementation.

## Appendix

Multimedia Appendix 1 Inclusion and exclusion criteria to the Risk Stratification and Early Alerting Regarding COVID-19 Hospitalization main study.

Multimedia Appendix 2 Risk Stratification and Early Alerting Regarding COVID-19 Hospitalization interview schedule.

## Abbreviations

ICF: informed consent form
QSG: QuickStart Guide
RiskSEARCH: Risk Stratification and Early Alerting Regarding COVID-19 Hospitalization
TDF: Theoretical Domains Framework
TUQ: Telehealth Usability Questionnaire
VCT: virtual clinical trial
