# Supplementary material for: Participant Experiences of a COVID-19 Virtual Clinical Study Using the Current Health Remote Monitoring Platform: Case Study and Qualitative Analysis
Source: JMIR Form Res. 2022 Jul 5;6(7):e37567. doi: 10.2196/37567 (PMC9258733; doi:10.2196/37567)
Supplement: Multimedia Appendix 1 [file formative_v6i7e37567_app1.pdf]

Multimedia Appendix. Inclusion/exclusion criteria to main RiskSEARCH study

Inclusion	Exclusion
Living in the United States (contiguous states)	Cannot confirm a positive PCR or antigen test for COVID-19
21 years or older	Unable to read English
Able to provide documentation of a positive PCR or antigen test for COVID-19 within the past 48 hours	Unwilling or unable to provide baseline data required for study entry
Self-reports comfortable and willing to wear the wearable device and interact with a tablet-based interface daily	Heavy tattooing on both upper arms (affected wearable sensor performance)
Able to provide a next of kin/designated person who can be contacted in the event of hospitalization for follow up	Known persistent atrial fibrillation (affected wearable sensor performance)
	Has taken/is taking part in a COVID-19 vaccine or treatment trial

This is a Multimedia Appendix to a full manuscript published in the J Med Internet Res. For full copyright and citation information see http://dx.doi.org/10.2196/jmir.37567
