# Supplementary material for: Participant Experiences of a COVID-19 Virtual Clinical Study Using the Current Health Remote Monitoring Platform: Case Study and Qualitative Analysis
Source: JMIR Form Res. 2022 Jul 5;6(7):e37567. doi: 10.2196/37567 (PMC9258733; doi:10.2196/37567)
Supplement: Multimedia Appendix 2 [file formative_v6i7e37567_app2.pdf]

Multimedia Appendix. RiskSEARCH Interview Schedule

Context

1. What led you to sign up for the study?

Study

2. We are interested in hearing about your views on the enrollment process, for example, navigating the website, completing the online eligibility questionnaire, talking to a study team member to give consent, getting the device and setting it up, etc. How was this process for you? (Prompt: barriers)
3. Are you still involved with/participating in the COVID-19 study? If not, why?
4. Have there been any negative effects of taking part in the study?
5. What did you think about the following COVID-19 study stuff?
    a. Website
    b. Emails from the study team
    c. Calls with the study team
    d. $100 for participating
    e. Other?
6. What was the best and the worst thing about taking part in the study?

Community by Current Health website

7. When you used the Community by Current Health website, could you tell me more about when and how often?
    a. How long did you use it for?
    b. If you stopped, why?
    c. What ideas do you have for encouraging people to keep using it?
8. Tell me about your experience using the website.
    a. Was the information helpful?
    b. Was the layout usable?
    c. What would you like to have seen?
    d. What were your patterns of use?
    e. Were there issues with the design?
9. Aside from this study, can you tell us about anything else you've used or done to contribute to COVID-19 research?

Wearable Device

10. Have you had any challenges in receiving, setting up the device or wearing it?
11. Have you had any technical issues?
    a. If so, have you received the support you needed to resolve the issues?
12. If you have had periods of time you have not worn the device, could you tell me why that is? (prompt: times of days or activities that led to removing the device)
13. What have you liked about wearing the device?

14. What have you disliked about wearing the device?
15. What could make using and wearing the device easier or more engaging?

Tablet

16. Have you had any challenges in using the tablet?
17. Have you had days you missed answering the survey and if so, what contributed to these?
18. What have you liked about the tablet?
19. What could make using the tablet easier to use or more engaging?
20. When setting up the kit, did you follow the printed guide, the on-tablet guide, or both?
21. Were the instructions on the tablet to fill in the questionnaire clear? Are there any improvements that we should consider?
22. Are there any other features you would like to see added to the tablet device?
23. We’re about to finish up. Now is your opportunity to share any other feedback or thoughts you have about the study.

This is a Multimedia Appendix to a full manuscript published in the J Med Internet Res. For full copyright and citation information see http://dx.doi.org/10.2196/jmir.37567
